# Association of soluble suppression of tumorigenicity 2 protein with new-onset atrial fibrillation in patients with acute ST-segment elevation myocardial infarction undergoing primary PCI

**DOI:** 10.3389/fcvm.2023.1207219

**Published:** 2023-09-21

**Authors:** Ting-ting Zhao, Tian-jiao Pan, Yi-bo Yang, Xiao-yang Pei, Yong Wang

**Affiliations:** ^1^ Department of Cardiology, Heping Hospital Affiliated to Changzhi Medical College, Changzhi, China; ^2^ Department of Day-Surgery, The First Affiliated Hospital of China Medical University, Shenyang, China; ^3^ Department of Cardiology, Shenzhen Luohu Hospital Group Luohu People’s Hospital (The Third Affiliated Hospital of Shenzhen University), Shenzhen, China

**Keywords:** soluble ST2, new-onset atrial fibrillation, acute ST-segment elevation myocardial infarction, PCI, predictor

## Abstract

**Background:**

Previous studies have indicated that the soluble suppression of tumorigenicity 2 protein (sST2) is associated with new-onset atrial fibrillation (NOAF) in patients diagnosed with coronary artery disease (CAD). However, the predictive value of sST2 in patients with acute ST-segment elevation myocardial infarction (STEMI) undergoing primary percutaneous coronary intervention (PCI) has not been well studied.

**Methods:**

A total of 580 patients with STEMI undergoing primary PCI were consecutively recruited between January 2021 and January 2023. These patients were then categorized into two groups: the NOAF group and the no NOAF groups based on the presence of NOAF during admission. The concentration of sST2 in blood samples was measured in all patients. The clinical data from the two groups were prospectively analyzed to investigate the predictive factors of NOAF in patients with STEMI undergoing primary PCI.

**Results:**

A total of 41 (7.1%) patients developed NOAF. The presence of NOAF has been found to be associated with various factors, including age, diabetes mellitus, hypertension, the left atrial (LA) diameter, N-terminal pro-brain natriuretic peptide, C-reactive protein (CRP), sST2, a Killip class of ≥2, and a final TIMI flow grade of <3. After including multiple factors, it was observed that LA diameter, CRP, sST2, a Killip class of ≥2, and a final TIMI flow grade of <3 remained significant risk factors for developing NOAF. The receiver operating characteristic (ROC) curve showed the following findings: (1) when the LA diameter exceeded 38.5 mm, the sensitivity and specificity values were observed to be 67.2% and 68.2%, respectively, and the area under the ROC curve (AUC) was 0.683 [95% confidence interval (CI): 0.545–0.732; *p* = 0.003]; (2) when the CRP level exceeded 8.59, the sensitivity and specificity values were observed to be 68.6% and 69.2%, respectively, and the AUC was 0.713 (95% CI: 0.621–0.778; *p* < 0.001); and (3) when the sST2 value exceeded 53.3, the sensitivity and specificity values were 79.2% and 68.7%, respectively, and the AUC was 0.799 (95% CI: 0.675–0.865; *p* < 0.001).

**Conclusion:**

sST2 has been identified as an independent predictor of NOAF in patients with STEMI undergoing PCI.

## 1. Introduction

Atrial fibrillation (AF) is one of the most common arrhythmias encountered in clinical practice. In fact, AF is prevalent in patients diagnosed with acute myocardial infarction (AMI). The incidence rate of new-onset AF (NOAF) ranges from 4.5% to 9.2% in patients with acute ST-segment elevation myocardial infarction (STEMI) undergoing primary percutaneous coronary intervention (PCI) (1–3). NOAF following STEMI is associated with an increased risk of in-hospital and long-term mortality (4, 5). With the coexistence of other cardiovascular risk factors, NOAF is associated with a higher risk of experiencing a nonfatal infarction (5), heart failure (6, 7), malignant arrhythmias (8), and stroke (4, 9). In addition, patients diagnosed with NOAF exhibit a higher tendency for experiencing a poor quality of life and incurring higher healthcare costs (10).

In patients with AMI, an inflammatory response occurs in the infarcted area, which is accompanied by myocardial dysfunction and mechanical stress. This process further facilitates the release of a soluble form of ST2 (sST2). As a member of the interleukin-1 receptor family, sST2 is associated with inflammatory responses and developing tissue fibrosis (11). The presence of inflammation and cardiac stress has been proven to induce structural and electrical remodeling and increase the risk of developing AF (12, 13). However, as a biomarker for fibrosis and inflammation, sST2 is not fully involved in the onset and development of AF, specifically in patients with STEMI undergoing primary PCI. Nortamo et al. (14) demonstrated that sST2 and high-sensitivity C-reactive protein (hs-CRP) exhibit a good predictive capacity for the occurrence of NOAF in patients with coronary artery disease (CAD), which may slightly improve the discrimination of the risk model. Chen et al. (15) suggested that sST2 is an independent predictor of NOAF in patients with AMI and can improve the accuracy of the AF risk model. Cardiac remodeling occurs in patients with STEMI undergoing PCI, and sST2 has been proven to be involved in the regulatory processes associated with cardiac remodeling. Furthermore, sST2 has demonstrated use in assessing the risk of adverse cardiac remodeling and the subsequent onset of heart failure in these individuals (16). Nevertheless, there is a lack of data on the relationship between sST2 and the risk of NOAF in patients with STEMI undergoing primary PCI.

Given the limited efforts in the prediction of NOAF in patients with STEMI undergoing primary PCI and the significant association between baseline sST2 levels and cardiac remodeling, we hypothesized that sST2 levels would be associated with the occurrence of NOAF in patients with STEMI undergoing primary PCI and may be useful to improve the prediction accuracy of NOAF in this patient population.

## 2. Methods

### 2.1. Study population

**Figure 1:**
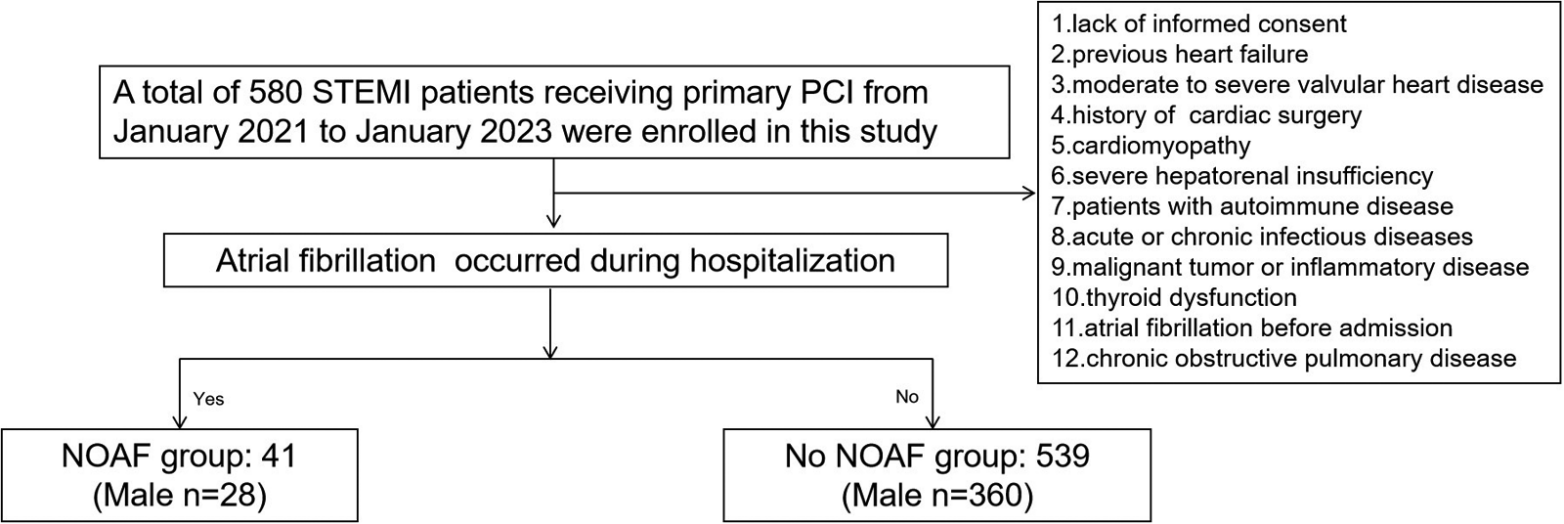
Patient enrollment of the study.

The study consecutively enrolled 612 patients diagnosed with STEMI undergoing PCI at Heping Hospital affiliated with Changzhi Medical College and Luohu People's Hospital from January 2021 to January 2023. The diagnosis of all patients was conducted in accordance with the criteria for STEMI established by the European Society of Cardiology (17). A primary PCI was performed on individuals who developed symptoms within 24 h. The exclusion criteria for this study include the following: (1) refusal to provide informed consent; (2) previous history of heart failure; (3) the presence of moderate to severe valvular heart disease; (4) prior history of cardiac surgery; (5) presence of cardiomyopathy; (6) severe hepatorenal insufficiency; (7) autoimmune disease; (8) presence of acute or chronic infectious diseases; (9) malignant tumor or inflammatory disease; (10) thyroid dysfunction; (11) history of AF prior to admission; and (12) a diagnosis of chronic obstructive pulmonary disease. Ultimately, a total of 580 participants were included in this study (Figure 1). This study was approved by the ethics committee of Heping Hospital, affiliated with Changzhi Medical College and Luohu People's Hospital, and was conducted in accordance with the principles of the Declaration of Helsinki. All patients provided informed consent prior to their inclusion in the study.

### 2.2. Clinical and laboratory data assessments

Following the hospitalization period, several clinical baseline data were recorded, including demographic characteristics, previous medical history, medication history, and echocardiographic and laboratory characteristics. Blood samples were collected via the median cubital vein after admission for laboratory analysis. The concentration of sST2 in blood samples was determined by fluorescent immunoassay and chromatography method employing a dry immunity analyzer (A2000, Emmy Medical Technology Co., Ltd., Guangxi, China). During admission, all patients were subjected to electrocardiogram (ECG) monitoring in the coronary care unit (CCU) for the first 3 days. In addition, 12-lead ECGs were conducted twice daily throughout the hospitalization period. For cases of suspected AF or to address a complaint of palpitations or arrhythmia, a 12-lead ECG was performed to confirm the presence of NOAF.

### 2.3. Interventional procedures

The radial artery was the preferred access site for all individuals. However, in certain patients, access was determined by the operators. PCI was performed following the guidelines for revascularization. The infarction-related artery was determined by at least two cardiologists. Angiographic and interventional procedure characteristics were recorded and analyzed. Written informed consent was obtained from all patients before the procedure.

### 2.4. Statistical analysis

The statistical analysis was performed using SPSS version 20.0 (IBM, USA). Normally distributed continuous variables were presented as the mean ± standard deviation and compared between groups using the Student's *t*-test. Data that were not normally distributed were expressed as medians and compared between groups using the Mann–Whitney *U* test. Categorical data were presented as rates or percentages and subjected to analysis using the chi-square test or Fisher's exact test. Univariable regression was performed to analyze the factors associated with NOAF, and multivariable logistic regression analysis was performed to determine the predictors of NOAF. The receiver operating characteristic (ROC) curve was used to evaluate the accuracy, sensitivity, and specificity of the left atrial (LA) diameter, sST2, and CRP in distinguishing NOAF from STEMI patients undergoing primary PCI, as well as the optimal cutoff value for predicting NOAF. All tests were two-sided, and *p*-values < 0.05 were considered significant.

## 3. Results

### 3.1. Baseline and clinical characteristics

A total of 580 patients with STEMI undergoing PCI were included in this study. Among this group, the occurrence of NOAF was observed in 7.1% (41/580) of the cases. Baseline characteristics, medications received in the hospital, and ECG and laboratory parameters are shown in Table 1. No significant differences were observed between patients with and without NOAF in terms of gender, current smoking status, alcohol use, dyslipidemia, stroke, previous history of PCI and AMI, family history of CAD, peripheral vascular disease, body mass index, or sleep apnea (*p* > 0.05). However, patients with NOAF tended to be older and exhibited a higher prevalence of comorbidities such as diabetes mellitus, hypertension, and cardiac dysfunction classified as Killip ≥2 (*p* < 0.05; Table 1). There were no differences in medications received during hospitalization, including angiotensin-converting enzyme inhibitors, angiotensin II receptor blockers, beta-blockers, statins, calcium channel blockers, and diuretics (Table 1). There were also no differences in terms of left ventricular end diastolic volume, left ventricular end systolic volume, or left ventricular ejection fraction. However, the diameter of the LA in patients with NOAF was significantly larger than that in patients without NOAF (32.6 ± 4.8 vs. 35.7 ± 6.5; *p* < 0.01; Table 1). In addition to creatine kinase, there were significant differences in clinical variables, including N-terminal pro-brain natriuretic peptide (NT-proBNP), CRP, and sST2, between patients with and without NOAF (*p* < 0.05; Table 1).

**Table 1:** Clinical characteristics of the study population.

Variables	No NOAF group (*n* = 539)	NOAF group (*n* = 41)	*p*-Value
Age, years	61 (42–78)	64 (45–79)	0.021
Gender (male), *n* (%)	360 (66.8)	28 (68.3)	0.84
Current smoker, *n* (%)	216 (40.1)	14 (34.1)	0.51
Alcohol use, *n* (%)	110 (20.4)	12 (29.3)	0.23
Diabetes mellitus, *n* (%)	226 (41.9)	25 (61.0)	0.02
Hypertension, *n* (%)	299 (55.5)	30 (73.2)	0.03
Dyslipidemia on admission, *n* (%)	188 (34.9)	16 (39.0)	0.61
Previous stroke, *n* (%)	32 (5.9)	4 (9.8)	0.31
Previous MI, *n* (%)	34 (6.3)	3 (7.3)	0.74
Previous PCI, *n* (%)	22 (4.1)	3 (7.3)	0.41
Family history of CAD, *n* (%)	102 (18.9)	10 (24.4)	0.41
Peripheral vascular disease, *n* (%)	43 (8.0)	5 (12.2)	0.37
BMI, kg/m^2^	24.5 ± 3.1	24.7 ± 1.9	0.65
Sleep apnea, *n* (%)	52 (9.6)	5 (12.2)	0.59
Killip ≥2, *n* (%)	102 (18.9)	16 (39.0)	<0.01
Medication history			
ACEI/ARB, *n* (%)	380 (70.5)	30 (73.2)	0.86
Beta-blocker, *n* (%)	322 (59.7)	26 (63.4)	0.74
Statins, *n* (%)	532 (98.7)	40 (97.6)	0.55
Calcium channel blockers, *n* (%)	141 (26.2)	15 (36.6)	0.15
Diuretics, *n* (%)	103 (19.1)	9 (22.0)	0.68
Echocardiographic analysis			
LA, mm	32.6 ± 4.8	35.7 ± 6.5	<0.01
LVEDV, mm	102.5 ± 22.0	106.8 ± 34.9	0.24
LVESV, mm	59.2 ± 15.6	63.5 ± 28.3	0.12
LVEF (%)	42.5 ± 6.0	42.2 ± 6.8	0.80
Laboratory analysis			
NT-proBNP, pg/mL	3,512.8 ± 5,883.3	5,487.7 ± 7,071.3	0.04
CRP, mg/L	7.6 ± 8.0	12.0 ± 7.3	<0.01
Creatine kinase, U/L	88.1 ± 48.4	84.0 ± 25.8	0.59
sST2, ng/mL	48.7 ± 23.6	71.5 ± 34.8	<0.01

MI, myocardial infarction; PCI, percutaneous coronary intervention; CAD, coronary artery disease; BMI, body mass index; ACEI, angiotensin-converting enzyme inhibitors; ARB, angiotensin II receptor blocker, LA, left atrium; LVEDV, left ventricular end diastolic volume; LVESV, left ventricular end systolic volume; LVEF, left ventricular ejection fraction; CRP, C-reactive protein; sST2, soluble ST2.

### 3.2. Angiographic and procedural characteristics of the studied patients

The angiographic and procedural characteristics of the studied patients are shown in Table 2. No significant differences were observed in relation to infarction-related arteries, multivessel disease, spontaneous coronary reperfusion, time from symptom onset to initial medical contact, reference diameter, or maximal stent length. However, patients with NOAF exhibited a higher proportion of final TIMI flow grade of <3 (31.7% vs. 17.6%; *p* = 0.03; Table 2).

**Table 2:** Angiographic characteristics of the studied patients.

Variables	No NOAF group (*n* = 539)	NOAF group (*n* = 41)	*p*-Value
Infarction-related artery			
LAD, *n* (%)	210 (39.0)	13	0.49
LCX, *n* (%)	129 (23.9)	9 (22.0)	
RCA, *n* (%)	200 (37.1)	19 (46.3)	
Multivessel disease, *n* (%)	201 (37.3)	15 (36.6)	0.93
Spontaneous coronary reperfusion, *n* (%)	105 (19.5)	9 (22.0)	0.70
Time from symptoms to FMC, min	358 (125–565)	358 (133–552)	0.64
Reference diameter, mm	3.2 ± 0.5	3.3 ± 0.5	0.48
Maximal stent length, mm	35.4 ± 16.7	34.0 ± 15.4	0.60
Final TIMI flow grade <3, *n* (%)	95 (17.6)	13 (31.7)	0.03

LAD, left anterior descending artery; LCX, left circumflex artery; RCA, right coronary artery; FMC, first medical contact; TIMI, thrombolysis in myocardial infarction.

### 3.3. Association of the markers with the risk of NOAF

**Figure 2:**
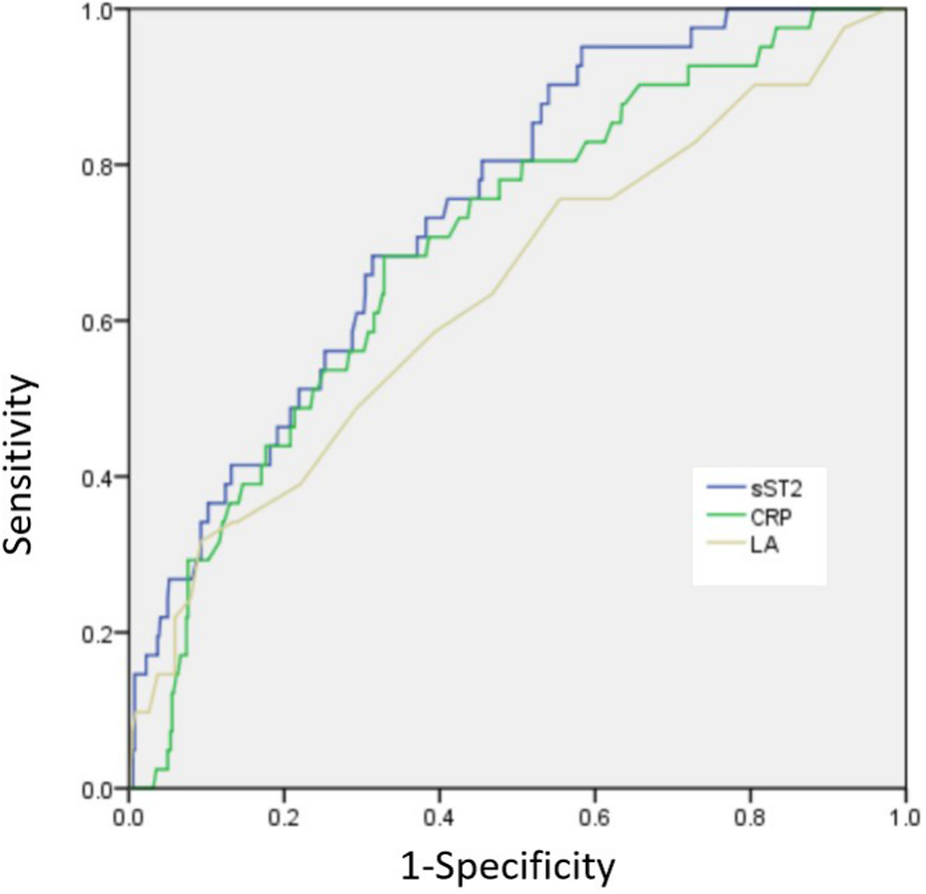
ROC curve showing the distinguishing ability of risk factors for the presence of NOAF.

The presence of NOAF was found to be associated with several factors, including age, diabetes mellitus, hypertension, LA diameter, NT-proBNP levels, CRP levels, sST2 levels, Killip class of ≥2, and a final TIMI flow grade of <3. After including multiple factors, it was found that LA diameter, CRP, sST2, Killip class of ≥2, and a final TIMI flow grade of <3 remained significant risk factors for developing NOAF (Table 3). The ROC curve showed the following findings: (1) when the LA diameter exceeded 38.5 mm, the sensitivity and specificity values were observed to be 67.2% and 68.2%, respectively, and the area under the ROC curve (AUC) was 0.683 (95% confidence interval [CI]: 0.545–0.732; *p* = 0.003); (2) when the CRP level exceeded 8.59, the sensitivity and specificity values were 68.6% and 69.2%, respectively, and the AUC was 0.713 (95% CI: 0.621–0.778, *p*<0.001); and (3) when the sST2 value exceeded 53.3, the sensitivity and specificity values were 79.2% and 68.7%, respectively, and the AUC was 0.799 (95% CI: 0.675–0.865; *p* < 0.001; Figure 2).

**Table 3:** Univariate and stepwise multivariate logistic regression analysis of NOAF.

	Univariate analysis			Multivariate analysis		
	OR	95% CI	*p-*Value	OR	95% CI	*p-*Value
Age, years	1.021	1.002–2.032	0.03	1.020	0.836–2.260	0.35
Alcohol use	1.121	1.011–1.821	0.02	1.112	0.895–1.835	0.49
Diabetes mellitus	1.184	1.011–2.120	0.04	1.180	1.021–1.925	0.52
Hypertension	1.229	1.027–1.624	0.04	1.220	1.019–1.620	0.29
LA, mm	1.992	1.251–7.608	0.03	1.902	1.220–7.672	0.03
NT-proBNP/1,000, pg/mL	1.268	1.001–2.032	0.03	1.201	0.895–1.994	0.42
CRP, mg/L	1.882	1.076–2.628	0.02	1.772	1.106–2.568	0.01
sST2, ng/mL	2.892	1.265–5.621	0.01	2.867	1.118–5.924	0.02
Killip ≥2	1.925	1.029–3.215	0.01	1.907	1.124–3.920	0.02
Final TIMI flow grade <3	1.792	1.102–5.621	0.02	1.780	1.129–5.391	0.02

LA, left atrium; CRP, C-reactive protein; sST2, soluble ST2; TIMI, thrombolysis in myocardial infarction.

## 4. Discussion

This study found that the incidence rate of NOAF in patients with STEMI undergoing PCI was 7.1%. LA, CRP, sST2, a higher proportion of Killip class of ≥2, and a final TIMI flow grade of <3 were independent predictors of NOAF in patients with STEMI undergoing PCI. sST2 may provide valuable information in relation to the discrimination risk associated with NOAF, surpassing the predictive capabilities of LA and CRP.

STEMI is a critical cardiovascular disease with high disability and mortality rates. Early reperfusion therapies were of vital importance for relieving symptoms and improving the prognosis. Primary PCI was the most widely used strategy in the revascularization of STEMI due to its simplicity, convenience, and efficiency. Despite the increasing prevalence of early PCI, AF remains a prevalent condition in patients with STEMI. Previous studies have suggested that the incidence rate of NOAF ranges between 4.5% and 9.2% in patients with STEMI undergoing primary PCI (1–3). NOAF following STEMI is associated with an increased risk of in-hospital and long-term mortality (4, 5). Although atrial fibrosis, inflammation, and cardiac stress have been suggested to increase the risk of AF (12, 13), the pathogenesis and clinical predictive biomarkers of NOAF in patients with STEMI undergoing primary PCI remain unclear.

The occurrence of NOAF in STEMI is still a common yet challenging issue in clinical practice. Further studies are required for the accurate prediction and appropriate management of this problem. Numerous studies have suggested that age, diabetes mellitus, and hypertension are independent risk factors for NOAF in patients with STEMI (2, 3). The present study found that patients with NOAF exhibited a higher mean age compared with those without NOAF. However, after multivariate adjustments, no statistically significant difference in age was observed between the groups. The potential reasons could be attributed to the inclusion of younger individuals and the presence of diverse races, comorbidities, and clinical characteristics, which may have influenced the observed outcomes. In addition, the number of included patients was relatively small. While a notable increase was observed in the incidence of diabetes mellitus and hypertension among patients with NOAF, no statistically significant difference was found after multivariate adjustments. We speculated that the current blood pressure and blood sugar control were not fully evaluated in this study. Consequently, our results may have to be interpreted differently compared with those in previous studies.

The LA diameter and its strain have been reported to be associated with NOAF (14, 18). Nortamo et al. (14) discovered that the LA diameter and NT-proBNP were independent predictors of NOAF in patients with CAD. However, the predictive value of the LA diameter in patients with STEMI undergoing primary PCI is still unknown. The present study found that the LA diameter exhibited a comparable and good predictive capacity in determining the risk of NOAF in patients with STEMI undergoing primary PCI. When the LA diameter exceeded 38.5 mm, the sensitivity and specificity values were 67.2% and 68.2%, respectively. As a biomarker of LA strain, higher NT-proBNP concentrations have been linked to the occurrence of NOAF in general population-based studies (19, 20). However, we found that the association of higher NT-proBNP concentrations with NOAF was lost after multivariate adjustments. Only specific STEMI patients were included in this study, which might have affected the results. Similar to a previous study, we found that a Killip class of ≥2 at admission was an independent predictor of NOAF in patients with STEMI undergoing primary PCI (2, 4, 21). Our study found that a postprocedural TIMI flow grade of <3 was an independent predictor of the occurrence of NOAF, which is similar to that found in a previous study (4). We speculated that patients exhibiting a TIMI flow grade of <3 would be associated with an increased risk of microvascular perfusion, a larger infarction size, and a higher risk of NOAF.

A previous study suggested that inflammation may play an important role in developing AF. Notably, patients with AF have obvious inflammatory changes in their atrial tissues (22). In addition, serum CRP levels are significantly higher in patients with paroxysmal and chronic AF (23). Furthermore, Chen et al. (15) suggested that hs-CRP is an independent predictor of NOAF in patients with STEMI undergoing PCI. CRP, as a biomarker of inflammation, is associated with an increased risk of developing NOAF (2). Similar to previous studies, we discovered that a higher CRP level was a predictive factor for an increased risk of NOAF in patients with STEMI undergoing PCI.

The presence of atrial fibrosis, inflammation, and cardiac stress can lead to structural and electrical remodeling, hence increasing the risk of developing AF (12, 13). AMI triggers an inflammatory response within the infarcted region, which promotes structural changes in the atrium, leading to the occurrence and development of NOAF. As a member of the interleukin-1 receptor family, sST2 is associated with inflammation and developing tissue fibrosis (11). The occurrence of STEMI leads to a significant increase in sST2 levels as a result of myocardial dysfunction and mechanical stress, which may activate IL-6, resulting in a higher level of TNF-α and hs-CRP from inflammatory and endothelial cells (24). This inflammatory reaction may lead to vascular endothelial injury, which in turn promotes the release of TNF-α and hs-CRP (25). This results in vicious cycles of inflammatory response, which may increase the risk of patients with STEMI developing NOAF. In addition, sST2 has been proven to exhibit a strong association with the process of extracellular matrix remodeling and the occurrence of inflammation, which has been suggested to be associated with arrhythmias (26). From another aspect, as STEMI occurs, early myocardial remodeling develops in both the left ventricle and the LA (27). The receptor ST2L plays a key role in preventing hypertrophy and fibrosis in tissues that suffer from mechanical strain. High concentrations of sST2 can prevent this protective effect and perhaps contribute to myocardial remodeling (28), increasing the risk for developing AF. As a biomarker of fibrosis, sST2 may play a potential role in both inflammatory processes and early myocardial remodeling in STEMI. Chen et al. (14) suggested that higher levels of sST2 were an independent predictor of NOAF in patients with AMI. Similar to a previous study, our findings suggest that sST2 is an independent predictor of NOAF in patients with STEMI undergoing PCI. In addition, Ma suggested that an elevated sST2 level may be involved in predicting the risk of emergency admission or heart failure in patients with AF. The exact mechanism of sST2 in AF among patients with STEMI undergoing primary PCI requires further investigation. However, as a promising biomarker for inflammation and fibrosis, sST2 exhibits several advantages over other biomarkers, primarily due to its low biological variability, minimal susceptibility to renal function, and favorable response to effective treatment. Our findings suggest that sST2 may serve as a new predictive biomarker for assessing the risk of developing NOAF in patients with STEMI undergoing primary PCI.

This study had some limitations. Firstly, this was a single-center study with a small sample size, which can lead to selective bias. Considering the paroxysmal and asymptomatic nature of AF, it is possible that AF cases could go undetected. Furthermore, the absence of screening measures to rule out preexisting AF raises the possibility that some of the patients classified as having NOAF may have really had AF prior to their admission for STEMI. Furthermore, the study design does not allow for the exclusion of chronically high sST2 levels, which may indeed contribute to increase the risk of NOAF; this is also a limitation of this study. Secondly, although multivariate analyses were performed, residual covariates may still be present; this may affect the predictive value of sST2. Thirdly, the major adverse cardiovascular and cerebrovascular events (MACCE) that occurred during hospitalization were not reported due to the relatively small sample size in the NOAF group. In addition, both short-term and long-term follow-up was not conducted. Finally, the number of patients included in this study was relatively small, and given the low event rates (40 patients presented with NOAF), the number of variables included in the multivariate analysis is quite large. This may have affected the statistical significance of the study. Future studies including a large sample size and involving multiple centers are needed to validate our conclusions.

## 5. Conclusion

The incidence rate of NOAF in patients diagnosed with STEMI undergoing primary PCI was found to be 7.1%. As a promising biomarker for inflammation and fibrosis, sST2 has been identified as an independent predictor of NOAF in patients with STEMI undergoing PCI.

## Data Availability

The raw data supporting the conclusions of this article will be made available by the authors, without undue reservation.
